# Crosstalk Between SMPDL3b and NADPH Oxidases Mediates Radiation-Induced Damage of Renal Podocytes

**DOI:** 10.3389/fmed.2021.732528

**Published:** 2021-09-29

**Authors:** Patrick Azzam, Marina Francis, Tarek Youssef, Manal Mroueh, Alaa Abou Daher, Assaad A. Eid, Alessia Fornoni, Brian Marples, Youssef H. Zeidan

**Affiliations:** ^1^Department of Anatomy, Cell Biology, and Physiology, Faculty of Medicine, American University of Beirut, Beirut, Lebanon; ^2^Peggy and Harold Katz Family Drug Discovery Center and Division of Nephrology, Department of Medicine, University of Miami, Miami, FL, United States; ^3^Department of Radiation Oncology, University of Rochester, Rochester, NY, United States; ^4^Department of Radiation Oncology, American University of Beirut Medical Center, Beirut, Lebanon; ^5^Baptist Health, Lynn Cancer Institute, Boca Raton, FL, United States

**Keywords:** ionizing radiation (IR), sphingolipids (SLs), reactive oxygen species (ROS), podocytes (MeSH: D050199), nephropathies, smpdl3b, NADPH oxidases (NOX)

## Abstract

Patients undergoing radiotherapy (RT) for various tumors localized in the abdomen or pelvis often suffer from radiation nephrotoxicity as collateral damage. Renal podocytes are vulnerable targets for ionizing radiation and contribute to radiation-induced nephropathies. Our prior work previously highlighted the importance of the lipid-modifying enzyme sphingomyelinase acid phosphodiesterase like 3b (SMPDL3b) in modulating the radiation response in podocytes and glomerular endothelial cells. Hereby, we investigated the interplay between SMPDL3b and oxidative stress in mediating radiation injury in podocytes. We demonstrated that the overexpression of SMPDL3b in cultured podocytes (OE) reduced superoxide anion generation and NADPH oxidase activity compared to wild-type cells (WT) post-irradiation. Furthermore, OE podocytes showed downregulated levels of NOX1 and NOX4 after RT. On the other hand, treatment with the NOX inhibitor GKT improved WTs' survival post-RT and restored SMPDL3b to basal levels. *in vivo*, the administration of GKT restored glomerular morphology and decreased proteinuria in 26-weeks irradiated mice. Taken together, these results suggest a novel role for NOX-derived reactive oxygen species (ROS) upstream of SMPDL3b in modulating the response of renal podocytes to radiation.

## Introduction

Radiation nephropathy is defined as an irreversible and detrimental renal injury caused by ionizing radiation. Kidneys are one of the most radiosensitive tissues, often receiving excessive exposure upon the management of abdominal or paraspinal tumors or total body irradiation. Subsequently, irrevocable intracellular cascades happen within the various renal structures, manifesting first as an acute phase of proteinuria to chronic kidney failure requiring dialysis or even kidney transplantation. The clinical manifestations of radiation nephropathy are presented as hypertension, azotemia, and severe anemia long-lasting after radiotherapy (RT) which culminates in renal failure. Histopathological features are also noted, with mesangiolysis, tubular atrophy, and tubulointerstitial scarring (1).

Ionizing radiation induces tissue injury through direct damage to the DNA structure, and indirectly via overproduction of reactive oxygen species (ROS) by water radiolysis. Data from numerous studies have suggested that late effects of RT are caused by an acute and chronic production of oxidative stress. Excessive ROS generation has deleterious consequences that initiate cascades of molecular events that disrupt signaling pathways and result in cellular damage. The latter happens mainly through the oxidation of major macromolecules such as proteins, lipids, and nucleic acid, abrogating their functions (2). Multiple physiological cellular sources mediate ROS production. Amongst these, the NADPH oxidases (NOX) family has received particular attention because of their normal function in host defense and cellular signaling including those in the kidney (3).

The mechanisms behind radiation-induced nephropathies are still largely unknown. The disorders identify the glomerulus as the main culprit in orchestrating the damaging phenotype through complex and dynamic interactions between glomerular, tubular, and interstitial cells. Among those, podocytes are highly specialized epithelial cells that wrap around capillaries to mediate glomerular filtration, a pivotal renal function which filters excess fluids and waste products into urine. Podocytes are identified as critical players in numerous kidney diseases such as focal segmental glomerulosclerosis (FGSG) and diabetic nephropathy, pointing to the crucial function that these cells fulfill in renal homeostasis. However, it remains ambiguous how radiation affects podocytes at a molecular level.

Recent studies conducted by our group and others reveal the importance of sphingolipids in mediating normal renal function especially in podocytes (4, 5). In this context, the sphingomyelinase phosphodiesterase acid-like 3b (SMPDL3b) enzyme was found to play a pertinent role in focal segmental glomerulosclerosis (6) and diabetic nephropathy (7), where SMPDL3b was found to mediate insulin receptor signaling (8). Although the intrinsic enzymatic activity of this protein remains to be elucidated, a growing body of evidence suggests a ceramide-1-phosphate (C1P) lyase-like activity (8, 9).

In this article, we hypothesized that radiation injury in podocytes is conducted through a crosstalk between SMPDL3b and NADPH oxidases. Radiation podocytopathy was mediated through a ROS-dependent mechanism initiated by NOXs that led to downregulated levels of SMPDL3b. Overexpression of the lipid-modifying enzyme mitigated NADPH oxidases activity post-RT and conferred radioprotection to podocytes.

## Materials and Methods

### Immortalized Human Podocytes Cell Culture, Irradiation and Treatment

Immortalized human podocytes, wild-type (WT) and SMPDL3b overexpressors (OE) were cultured on collagen-coated dishes and differentiated in RPMI-1640 medium containing 10% FBS (Sigma-Aldrich) and 5% penicillin/streptomycin (Biowest). Briefly, cells were propagated at 33°C in 1% insulin-transferrin-selenium 100x (Gibco, USA) containing media on T25 flasks and then thermoshifted for differentiation for 14 days at 37°C. A single dose of 8Gy was delivered from an RS2000 X-ray irradiator (225 kV) according to the manufacturer's specifications (Rad Source Technologies, Suwanee, GA, USA). The dose rate was adjusted to 265 cGy/min. Cells were then irradiated (8Gy) and treatment was stopped by removing the media and adding cold saline solution at the proper time points. For ROS scavenging and NOX1/4 inhibition, cells were treated with 100 μM of N-acetylcysteine (NAC) and 10 μM GKT137831 dissolved in DMSO and PBS respectively for 2 hours prior to irradiation. An equal quantity of DMSO or PBS was added to the control samples.

### Immunofluorescence With DHE and DAPI

Superoxide anions were detected using dihydroethidium (DHE) stain by quantification of mean immunofluorescence (MIF). Briefly, cells were grown on 35 mm dishes and were stained with 5μM of DHE for 1 hour at 37°C, fixed with 4% of formaldehyde for 20 minutes, and stained with DAPI. Podocytes were visualized using Zeiss confocal microscope (LSM710 Meta, Carl Zeiss, Inc., Thornwood, NY, USA). Data were analyzed using the LSM Image Browser Software.

### Quantitative RT-PCR

Cells were washed with ice-cold PBS and then scrapped from the plate with Trizol for RNA extraction. RNA was quantified by NanoDrop (Thermo Fischer Scientific) and converted to cDNA using iScript cDNA kit (Bio-Rad). cDNA was then diluted (1:10) and 2 μL were added per 20 μL of reaction. Using the iTaq Syber Green, the reaction was executed in real-time PCR system (CFX384 Touch Real-Time PCR Detection System, Bio-Rad, USA) as per the manufacturer′s instructions. Real-time and qualitative PCR was done for human NOX1 (F:5′-GCAGGGAGACAGGTGCCTTTTCC-3′; R: 5′-CTACAGACTTGGGGTGGGAGGT-3′), NOX2 (F:5′-TTCCAGTGCGTGCTGCTCAACA-3′; R:5′-CTGCGGTCTGCCCACGTACAA-3′), NOX3 (F:5′-CCATCCATGGGACGGGTCGGA-3′; R:5′-AGGGGTGCCACTCCAGCGAA-3′), NOX4 (F:5′-CTGGCTCGCCAACGAAGGGG-3′; R:5′-GCTTGGAACCTTCTGTGATCCTCGG-3′), NOX5 (F:5′-GGAGCAAGGTGTTCCAGAAAG-3′; R:5′-AAGGCTCCTCCAAGTAGCAAG-3′), SMPDL3b (F:5′-GCATGGTTCCGGGAGGGCTT-3′; R5′-TGCCCGAAGAACTGCCCTGC-3′), GAPDH (F:5′-TGCACCACCAACTGCTTAGC-3′; R:5′-GGCATGGACTGTGGTCATGAG-3′) and B-actin (F: 5′GCATGGGTCAGAAGGATTCCT-3′; R: 5′-TCGTCCCAGTTGGTGACGAT-3′).

### MTT Assay

MTT kit (Abcam) was applied as per the manufacturer's recommendation. Briefly, cells were seeded at 15,000 cells/well on collagen-coated 24-well plates and incubated for 24 hrs at 33°C. Afterward, cells were shifted for differentiation at 37°C, and N-acetylcysteine or GKT pretreatment was administered as previously described before irradiation. The MTT assay was applied at 24 hrs post-irradiation. A microplate reader (Multiskan EX, Thermo-Fisher Scientific) was used to measure the absorbance at 590 nm by spectrophotometry.

### Protein Extraction and Western Blotting

Podocytes were homogenized in cold RIPA buffer (150 mM NaCl, 1% NP-40, 0.5% sodium deoxycholate, 0.1% sodium dodecyl sulfate, and 50 mM Tris pH 8) supplemented with 10 μL of protease and phosphatase inhibitor cocktail each (Biowest). Protein lysates were collected after centrifugation at 13500 rpm, for 30 min at 4°C. Protein quantification was done using a Lowry Reagent Assay kit from Bio-Rad. Samples were then prepared after quantification with 2X Laemmli sample buffer (Bio-Rad). An equal number of proteins (25-40 μg) were then loaded into 10-12-15% SDS-PAGE gels (Bio-Rad) and transferred on nitrocellulose membrane for 2hrs on ice at 300mA. Membranes were then blocked with 5% skimmed milk or BSA in Tris-saline solution for 1 hour at room temperature. The following primary antibodies were used, each according to the protocol suggested by the manufacturer: rabbit polyclonal anti-SMPDL3b (1:1000) (Genway Biotech, Inc., San Diego, CA, USA), mouse monoclonal anti-GAPDH (1:1000) (Abcam), rabbit monoclonal NOX1 and NOX4 antibodies (1:500) (Abcam), rabbit monoclonal caspase 3 (1:250) (Cell Signaling). Membranes were incubated with the primary antibodies overnight then washed 3 times for 10 minutes each in Tris-saline solution with 0.1% Tween 20. Horseradish peroxidase-conjugated secondary antibodies were used, and the images were developed using enhanced chemiluminescence (Bio-Rad). Densitometry was performed using the ImageJ software (National Institute of Health, Bethesda, MD, USA).

### NADPH Oxidase Assay

The activity of the NADPH oxidases enzymes was assessed in cultured podocytes as previously described (10). Cells were washed with ice-cold PBS and scraped from the plate with a special lysis buffer (20 mM KH2PO4 pH 7.0, 1 mM EGTA, 10μL Protease Inhibitor). The homogenate was quantified using the Bio-Rad protein assay reagent. The assay was conducted on 25μg of homogenates which were added to 50 mM phosphate buffer (pH 7.0) containing 1 mM EGTA, 150 mM sucrose, 5 μM lucigenin, and 100 μM NADPH. Light emission was measured after 30 seconds for 8 minutes in a luminometer. The first and last readings were discarded, and a buffer blank was subtracted from each reading. Superoxide production was averaged and expressed as relative light units/min.mg of protein.

### Animal Studies

To assess podocyte cell damage in vivo, ten weeks old C57BL6 male mice were treated with normal saline or GKT137831 with or without a single dose of 14 Gy. GKT137831 treatment was administered by oral gavage 1 hr prior to irradiation at a prophylactic concentration of 20 and 40 mg/kg 1hr following irradiation. Mice were then sacrificed at 24 hrs or 26 weeks post-radiation. 26 weeks has been chosen as the time for phenotype analysis to assess late radiation sequelae (1, 11, 12) Both kidneys were harvested and processed for histological immunohistochemical and molecular studies. Mice were irradiated using RS2000 X-ray irradiator (225 kV) in a specialized lead jig that precisely delivered stereotactic doses to the kidneys. Mice were anesthetized with ketamine/xylazine (1:5) at a concentration of 80 and 8 mg/kg respectively.

### Morphometric Glomerular Assessment and Histology

Right kidney was removed for histological analysis and the left kidney was collected for glomeruli extraction. Hematoxylin-eosin (H&E) and Masson Trichrome staining of paraffin-embedded kidney sections (5 μm thick) were performed using a standard protocol. Histological images were visualized using a light microscope (Olympus BX 41, Tokyo, Japan) at 20x magnification. Glomerular area surface was analyzed via ImageJ software. Twenty glomeruli per section were analyzed for collagen deposition by quantitating positive areas for Masson Trichrome staining along with morphometric glomerular parameters, both performed by two blinded independent investigators.

### Blood Pressure Measurement

Blood pressure was measured daily by non-invasive determination of tail blood volume, flow, and pressure using a volume pressure-recording sensor and an occlusion tail-cuff (CODA System; Hakubatec Lifescience Solutions, Tokyo, Japan). This is a highly accurate system with the capability of measuring systolic and diastolic blood pressures and heart rate simultaneously and non-invasively. Before measurement, the mice were placed on a 37°C warming pad until the temperature of the tail region reached 37°C according to an infrared thermometer. Following warming, the mice were trained for 15-minute sessions each day for 7 days or until we obtained stable blood pressure recordings.

### Statistical Analysis

Results were expressed as the means ± SEM. One-way or Two-way ANOVA along with t-test were used to compare groups, and results were considered statistically significant if *P* < 0.05 (GraphPad Prism software; La Jolla, CA, USA). Results shown are the mean SEM values of at least three independent experiments.

## Results

### Overexpression of SMPDL3b Decreases Radiation-Induced Superoxide Anion Generation in Podocytes

**Figure 1:**
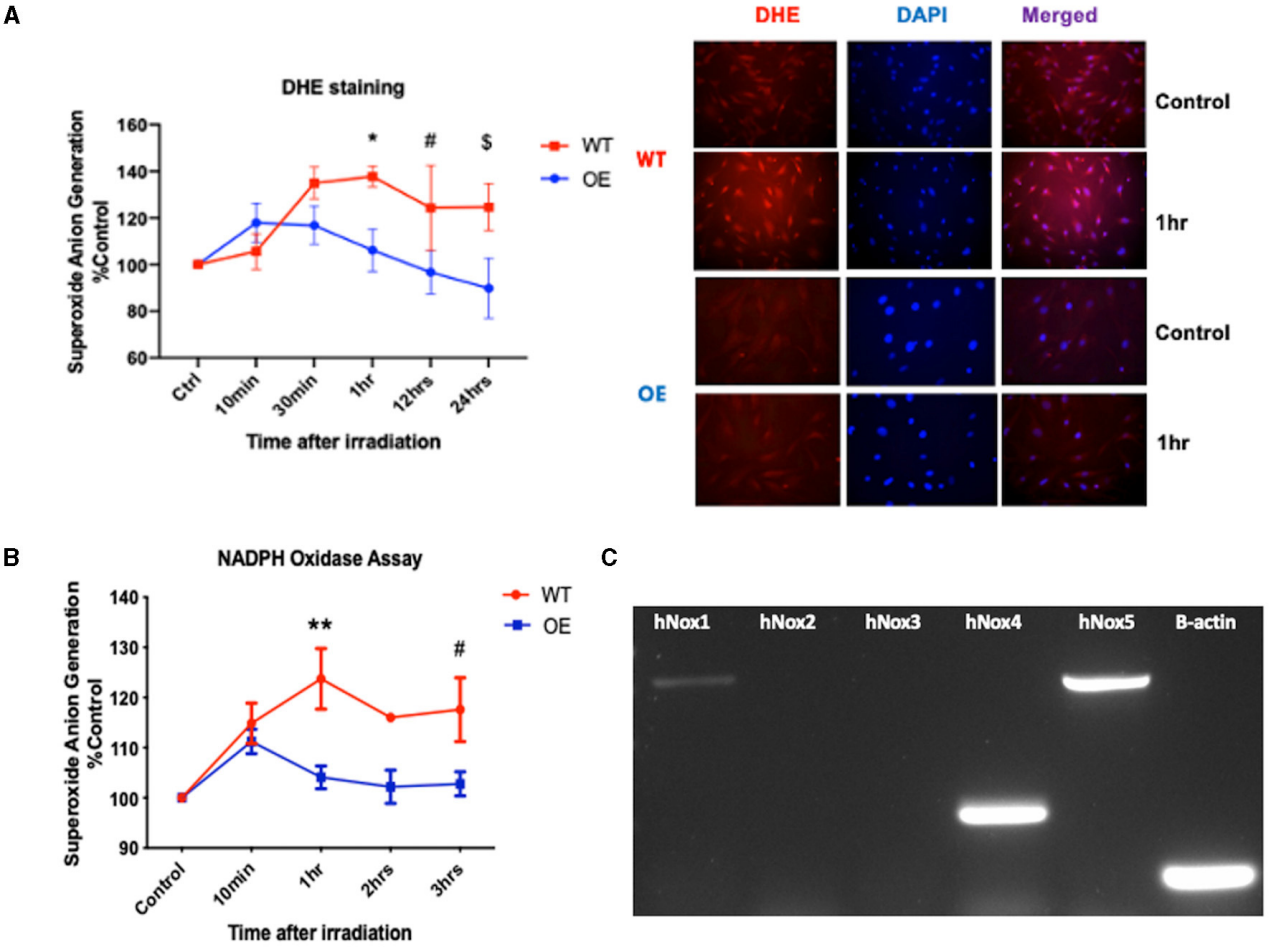
Overexpression of SMPDL3b protects podocytes from radiation-induced reactive oxygen species. **(A)** Quantification of DHE mean immunofluorescence (left panel) at baseline (control), 10 and 30 minutes, 1hr, 12hrs, and 24 hours post-irradiation at 8 Gy (WT vs OE: *p = 0.007 at 1hr, #*p* = 0.0156 at 12 hrs, $*p* = 0.0039 at 24hrs). Immunofluorescence staining with DHE and DAPI of podocytes (right panel) at baseline 0 Gy (control) and 8 Gy at 1hr post-irradiation. Objective 20x. **(B)** Superoxide anion generation measured via lucigenin mediated NADPH oxidase assay in both WT and OE human podocytes at baseline (control) and 10min, 1hr, 2hrs, 3hrs after 8 Gy irradiation (WT vs. OE: **p = 0.0036 at 1hr, #*p* = 0.0340 at 3hrs). **(C)** Conventional PCR analysing expression of NOX1 (730bp),−2,−3,−4 (253bp), and−5 (762bp), ß-actin (106bp) in WT human podocytes cell lysate. The results shown are the mean values of at least three independent experiments.

Our prior work uncovered the differential expression of phosphorylated histone H2AX (γ-H2AX) between the wild-type (WT) and SMPDL3b overexpressing (OE) human podocytes cell lines. This finding suggested a radioprotective role for SMPDL3b against DNA damage (13). In fact, reactive oxygen species (ROS) are among the primary causes of DNA insults following low linear energy transfer (LET) ionizing radiations, such as those used in conventional clinical radiotherapy. ROS are generated from radiation-induced water radiolysis and predominantly contributed to the formation of DNA double-strand breaks (DSBs). Passage through mitosis with unresolved radiation-induced DNA DSBs leads to cell death. The induction of DNA DSBs leads to phosphorylation of H2AX (γ-H2AX), and γ-H2AX foci are widely used as a DNA damage marker (4, 14). Thus, we investigated the potential interplay between SMPDL3b and ROS generation. To that end, we quantified the superoxide anion generation via DHE staining of both cell lines at different time points after irradiation (Figure 1A). OE podocytes showed a substantial decrease in the superoxide anion generation compared to WT starting at 1 hr post-irradiation.

### Differential Expression of NOX1/NOX4 Is Observed Between OE SMPDL3b and WT Podocytes

NADPH oxidases are one of the main sources implicated in the generation of superoxide anions (3). We investigated the differential expression of NADPH oxidases in the two cell lines. For that purpose, we performed the NADPH oxidase assay to assess NOXs' enzymatic activity following irradiation. Our data demonstrated a differential increase in NADPH oxidase activity in WT podocytes compared to OE especially at 1hr post-irradiation (Figure 1B). Following conventional PCR (Figure 1C), we confirmed the detection of NOX1 (730bp), NOX4 (253bp), and NOX5 (763bp) mRNAs in our human podocytes' cell lines. This is consistent with prior studies confirming NOX1 and NOX4 as the major NOX isoforms expressed in podocytes (3).

**Figure 2:**
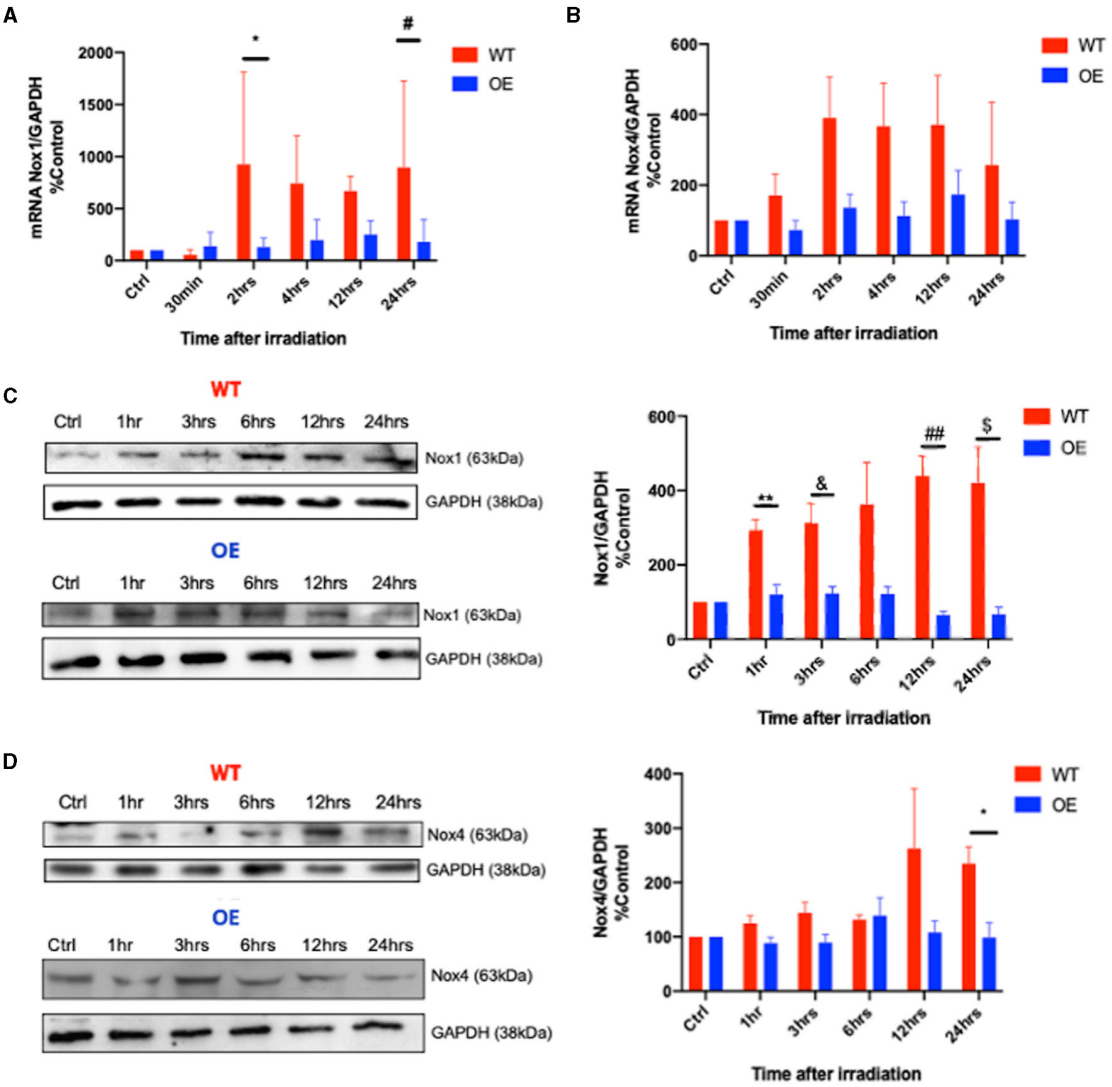
Differential expression of NOX1 and NOX4 in WT and OE podocytes upon radiation. Transcriptional RT-PCR analysis of NOX1 **(A)** (*p = 0.007 for WT vs. OE at 2 hrs, #*p* = 0.0142 at 24hrs) and NOX4 **(B)** mRNA expression in WT and OE human podocytes at baseline, 30 min, 2 hrs, 4 hrs, 12 hrs, and 24 hrs after 8 Gy irradiation. Translational immunoblotting analysis of NOX1 **(C)** (WT vs. OE: 1hrs **p < 0.0041; 3 hrs &*p* < 0.0303; 12 hrs ##*p* < 0.0052; 24hrs $*p* < 0.0321), and NOX4 **(D)** (WT vs. OE: 24 hrs *p < 0.0245) in WT and OE human podocytes at baseline, 1 hr, 3 hrs, 6 hrs, 12 hrs, 24 hrs following 8 Gy irradiation. The results shown are the mean values of at least three independent experiments.

Next, we examined whether radiation induces a differential NOX1/NOX4 expression in both cell lines. NOX1 mRNA levels in WT cells showed a time-dependent increase in contrast to OE cells (Figure 2A). A similar trend was observed in mRNA levels of NOX4 between both cell lines (Figure 2B). Comparable results were also found in NOX1 and NOX4 protein levels (Figures 2C,D).

### Inhibition of NOX1/NOX4 via GKT137831 Restores SMPDL3b's Levels in WT Podocytes

**Figure 3:**
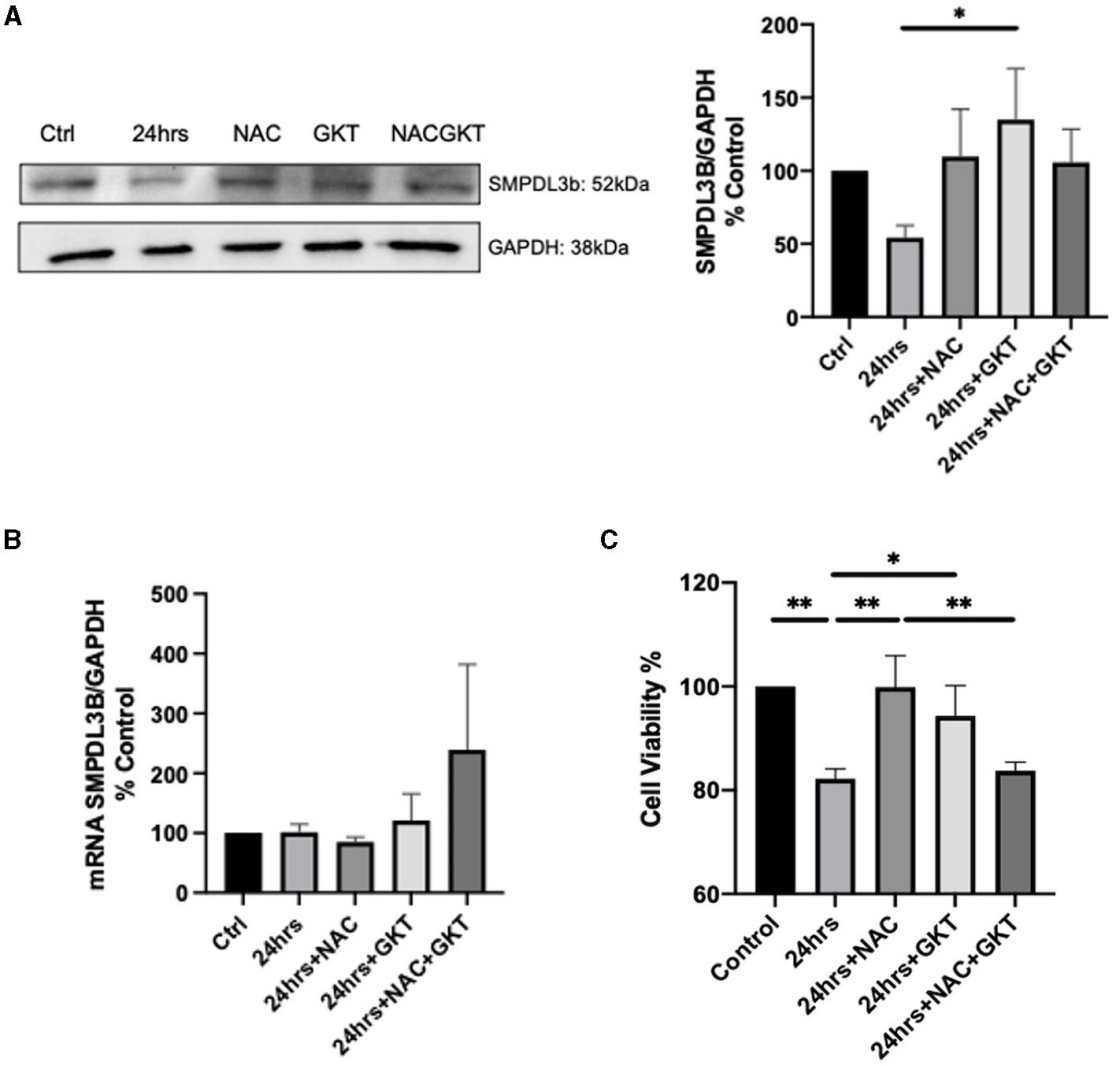
SMPDL3b protein levels are restored upon administration of both NAC and GKT after 24 hours of radiation in podocytes. SMPDL3b protein **(A)** (24 hrs vs. 24 hrs+GKT: *p = 0.0382) and mRNA expression **(B)** at baseline and 24 hrs after 8 Gy irradiation in pre-treated WT podocytes with ROS scavenger N-acetylcysteine (100 μM) and/or dual NOX1/4 inhibitor GKT (10 μM). **(C)** Survival profile of WT podocytes at baseline and 24hrs post-irradiation pre-treated with NAC and/or GKT via MTT assay detected by spectrophotometry (Ctrl vs. 24 hrs **p = 0.0045; Ctrl vs. 24 hrs+NAC+GKT **p = 0.0083; 24 hrs vs. 24 hrs+NAC **p = 0.0047; 24 hrs vs. 24 hrs+GKT *p = 0.0401; 24 hrs+NAC vs. 24 hrs+NAC+GKT **p = 0.0088). The results shown are the mean values of at least three independent experiments.

These results led us to investigate the relationship between SMPDL3b and NADPH oxidases. To that end, we used the ROS scavenger N-acetylcysteine (NAC), and GKT137831, a dual inhibitor of NOX1 and NOX4. It has been previously demonstrated that SMPDL3b starts to decrease 4hrs post-irradiation in WT podocytes (13). Interestingly, administration of both NAC and GKT restored protein levels of SMPDL3b 24hrs following irradiation while it did not affect its transcriptional levels (Figures 3A,B). This was associated with improved cell viability as measured using MTT assay (Figure 3C).

### Early and Late Radiation Sequelae Ameliorated Upon GKT Treatment in Irradiated C57BL6 Mice

**Figure 4:**
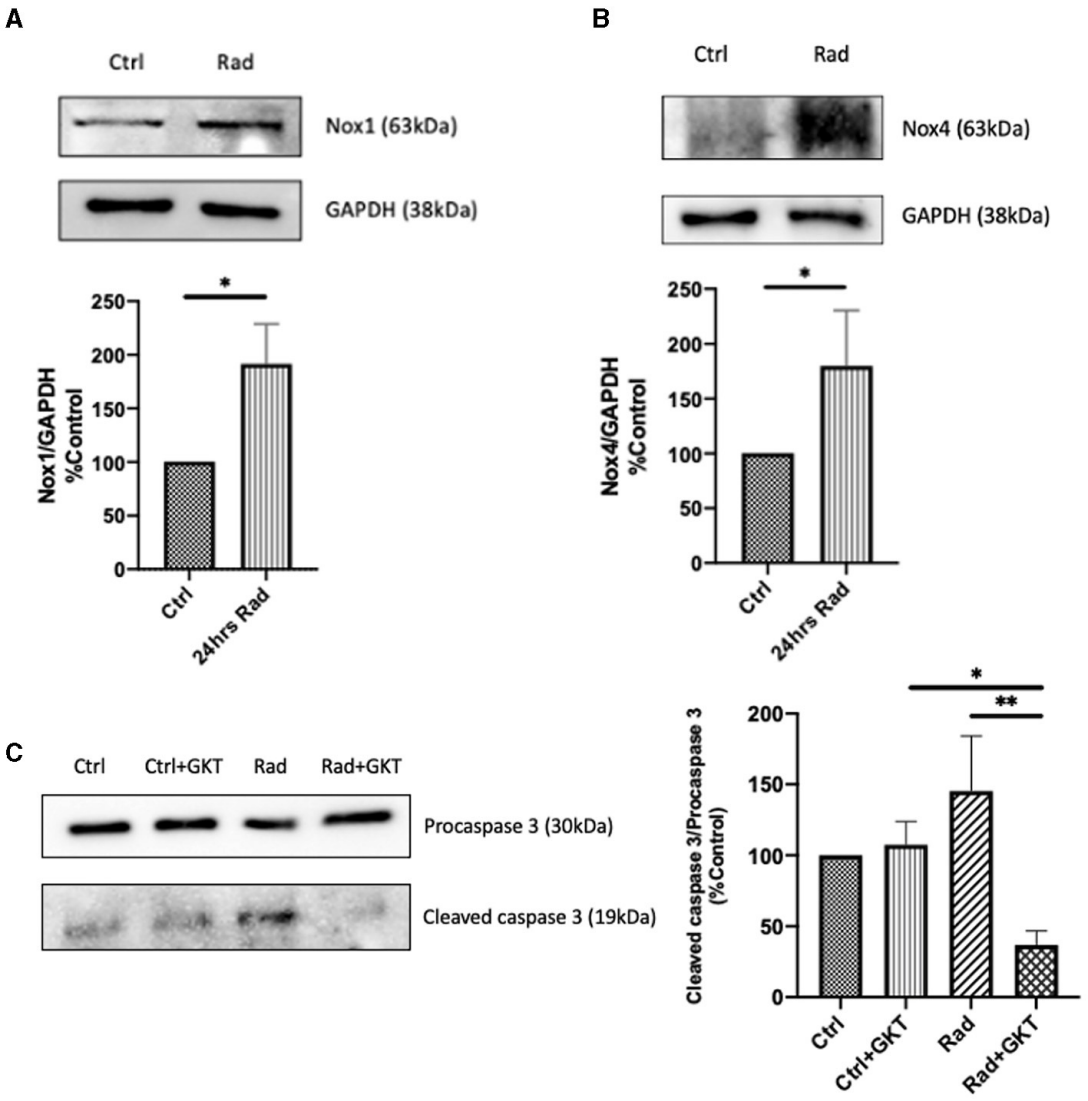
Radiation causes acute upregulation of NADPH oxidases protein expression in kidneys of 24hrs post-RT C57BLC/6 mice. Increase of protein levels of NOX1 **(A)** (Ctrl vs. Rad *p = 0.0493), and NOX4 **(B)** (Ctrl vs. Rad *p = 0.0196) detected by immunoblotting in renal cortices at 24hrs post-irradiation. Post-irradiation cleavage of caspase 3 reduced upon administration of GKT (Ctrl+GKT vs. Rad+GKT *p = 0.0498, Rad vs. Rad+GKT **p = 0.0075) **(C)**. The results shown are the mean values of at least three independent experiments.

Next, we proceeded to verify our results via an *in vivo* model. C57BL6 males at 10 weeks of age were treated with either normal saline or a full dose of GKT (40 mg/kg) 1 hr prior to focal renal radiation of 14 Gy. All mice were sacrificed 24 hours later to assess the acute radiation effects. A notable increase of protein expression of NOX1 and NOX4 was identified in the irradiated group compared to control littermates (Figures 4A,B). Furthermore, inhibition of NOX1/4 via GKT treatment in the irradiated group decreased cleavage of caspase 3, indicating improved survival at a cellular level (Figure 4C).

**Figure 5:**
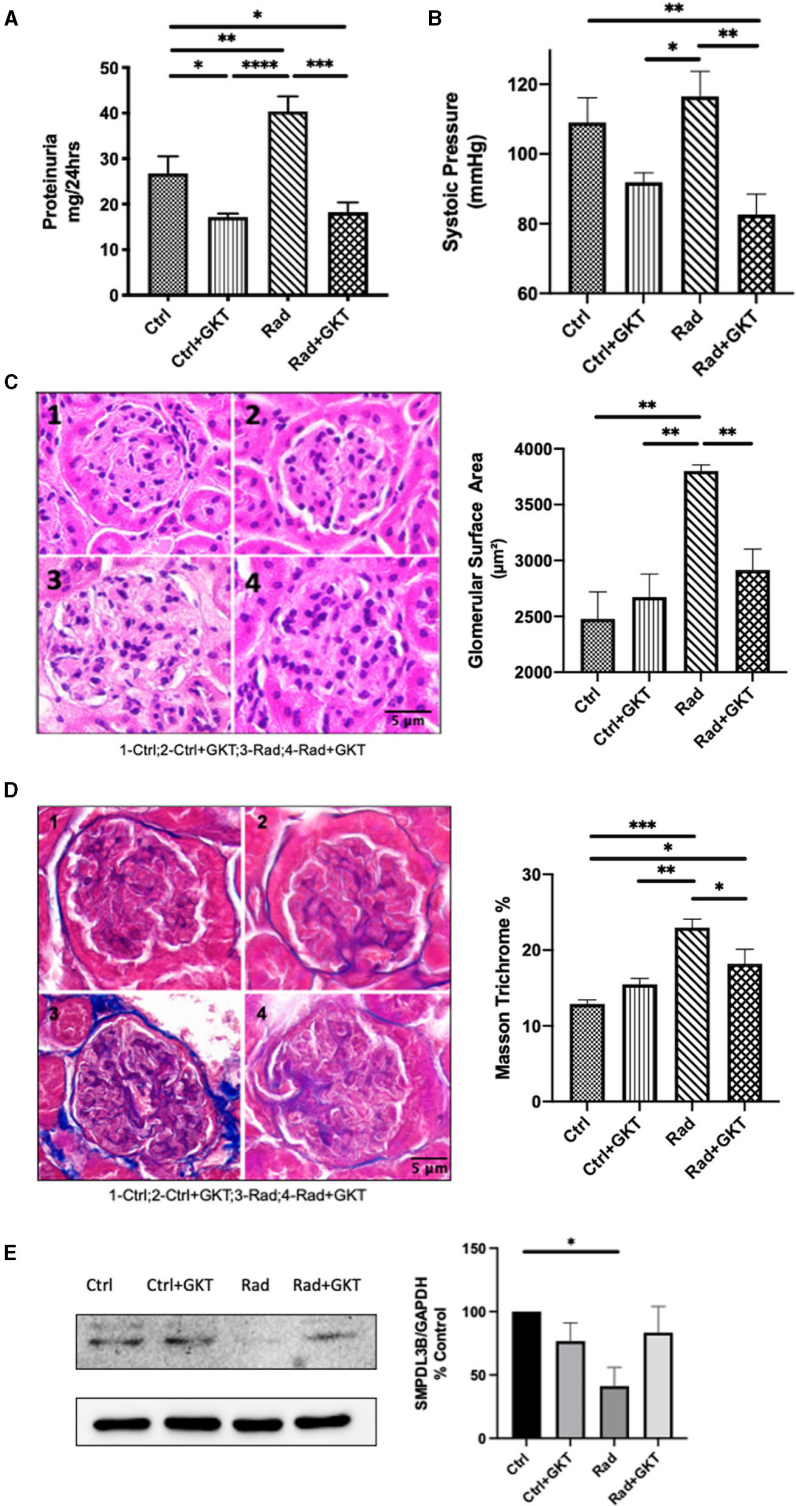
Administration of GKT restores radiation injury markers in 26 weeks post-irradiation mice. **(A)** Proteinuria measured in C57BLC/6 mice at 22 weeks post-RT by urine collection (Ctrl vs. Ctrl+GKT *p = 0.0308; Ctrl vs. Rad **p = 0.0452; Ctrl vs. Rad+GKT *p = 0.0484; Ctrl+GKT vs. Rad ****p < 0.0001; Rad vs. Rad+GKT ***p = 0.0001). **(B)** Systolic pressure measured by non-invasive tail-cuff method (Ctrl vs. Rad+GKT **p = 0.0089; Ctrl+GKT vs .Rad *p = 0.0134; Rad vs. Rad+GKT**p = 0.0018). GKT treatment ameliorates partially morphological parameters of irradiated glomeruli in mice. Representative photomicrographs of glomeruli from Ctrl (1), Ctrl+GKT (2), Rad (3), Rad+GKT (4) kidneys harvested from C57BLC/6 mice at 24 weeks post-radiation; Quantification of all parameters of at least 20 glomeruli in all groups **(C)** Paraffin-embedded sections, 5 μm thick, were stained with H&E (20x), scale bar 5 μm (Ctrl vs. Rad **p = 0.0010; Ctrl+GKT vs. Rad **p = 0.0027; Rad vs. Rad+GKT **p = 0.0099). **(D)** Paraffin-embedded sections, 5 μm thick, were stained with Masson Trichrome (blue stain) for collagen deposits (20x), scale bar 5 μm (Ctrl vs. Rad ***p = 0.0008; Ctrl vs. Rad+GKT *p=0.0222; Ctrl+GKT vs. Rad **p=0.0055; Rad vs. Rad+GKT *p=0.0347). **(E)** Immunoblotting of SMPDL3b in renal cortices homogenates of 26 weeks irradiated and non-irradiated C57BL6 mice. SMPDL3b is restored upon GKT treatment (Ctrl vs. Rad *p = 0.0214; Rad vs. Rad+GKT ns *p* = 0.0724). The results shown are the mean values of at least three independent experiments.

We were also interested in investigating late radiation sequelae. C57BL6 males of 10 weeks were treated with either normal saline or a prophylactic dose of GKT (20 mg/kg) 1 hr before focal renal radiation of 14 Gy. Following X-ray exposure, those who received a prophylactic dose were further treated with a full 40 mg/kg of GKT. All mice were sacrificed 26 weeks post-irradiation. Significant proteinuria was noted in the irradiated group compared to control, which was restored to normal levels upon administration of GKT (Figure 5A). High levels of systolic pressure were detectable in the irradiated group and alleviated in GKT treated littermates (Figure 5B).

To assess the impact of ionizing radiation on renal morphology, kidney sections were stained with hematoxylin and eosin. Quantification demonstrated irradiation-induced hypertrophy of glomeruli as evidence by increased glomerular area surface in irradiated mice which was partially alleviated upon administration of GKT (Figure 5C). We observed thinning of parietal cells of Bowman's capsule and capillary dilatation in glomerular tufts that were restored with GKT administration. Additionally, collagen staining increased in irradiated glomeruli which was downregulated with GKT treatment (Figure 5D). Furthermore, protein levels of SMPDL3b were downregulated in 26 weeks post-irradiated renal cortices homogenates with a tendency of being restored upon GKT treatment (Figure 5E). This is in line with a previous study of ours where SMPDL3b was decreased in 11 weeks of irradiated mice glomeruli (13).

## Discussion

Radiation nephrotoxicity remains a clinical concern and can be an obstacle to treatment in some cancer patients undergoing RT, especially those with compromised renal function. Despite advances in delivering precise radiation doses to the tumor, some normal tissues are unavoidably irradiated and other out-of-target normal tissues also receive a dose that results in acute and late RT side effects. Therefore, there is a pressing clinical need to investigate the molecular events underlying these acute and late normal tissue effects.

SMPDL3b was shown to play a protective role in a panel of diseases. Pioneering work suggested that treatment of patients with rituximab at the time of kidney transplant might prevent recurrent FSGS by modulating podocyte function in an SMPDL3b–dependent manner (6). Prior work has shed light on the radioprotective role of SMPDL3b in cultured podocytes. OE podocytes showed a reduced number of γ-H2AX foci. Moreover, these OE cells had abrogated actin cytoskeleton remodeling and caspase 3 cleavage post-RT, in contrast with WT cells. Intriguingly, SMPDL3b exhibited a time-dependent decrease following irradiation both *in vitro* and 3 months post-RT *in vivo* (13). Nevertheless, the molecular mechanisms underlying these events were not clear.

The current study examines the relationship between SMPDL3b and oxidative stress in the context of RT in podocytes. The novelty of our study suggests a crosstalk between SMPDL3b and NADPH oxidases. In wild-type cells, irradiation increased NOXs expression and enzymatic activity and thus upregulated ROS production. The administration of NAC and/or GKT restored levels of SMPDL3b, suggesting that NOX-derived ROS may account for the time-dependent decrease of the lipid-modifying enzyme after RT. Alternatively, overexpression of SMPDL3b led to decreased enzymatic activity and levels of NADPH oxidases, confirming a crosstalk.

It is plausible that SMPDL3b could be degraded in an oxidative-dependent manner, initiated by NOXs. In fact, protein oxidation facilitates both proteasomal and lysosomal-mediated degradation (15) and it remains ambiguous which pathway is involved in the loss of the enzyme. There are currently no known ROS-dependent modifications in the protein's configuration. Proteasomes and lysosomes which are the main cellular compartments responsible for proteolysis, are redox-sensitive (15) and could be activated by ionizing radiation to eliminate potentially damaging oxidized proteins (16, 17). Further experiments are warranted to investigate the possibility of redox modifications of SMPDL3b. The latter could be examined through recent omics-based approaches such as thiol redox proteome (18, 19). Thiol is a redox-sensitive group that allows cysteine to be subjected to all kinds of oxidative reactions. A growing body of evidence suggests that thiol redox modifications are not random cellular incidents but well-organized and coordinated events leaving a particular signature on the oxidized molecule.

Overexpression of SMPDL3b conferred cellular protection by alleviating radiation-induced ROS generation. Nevertheless, the mechanism behind this inhibition remains to be established. Emerging evidence suggested a C1P-lyase like activity for SMPDL3b (8, 9). Following that, we can speculate that the overexpression of the lipid-modifying enzyme leads to a shift in the lipidomic profile of the podocytes. Consequently, as second messengers and bioactive entities, sphingolipid metabolites play an essential role in regulating biological processes and might potentially alter signaling pathways (20, 21). Previous data analysis of mass spectrometry showed that irradiated OE SMPDL3b podocytes had downregulated levels of ceramide-1-phosphate, while interestingly, no changes in sphingosine and ceramides were noted, (8, 13). On the other hand, irradiated WT podocytes exhibited upregulation in ceramides and a decrease in sphingosine. Ceramides were shown to activate ROS generating entities like NADPH oxidases and to form lipid-rafts that assemble the corresponding subunits. On the other hand, sphingosines were found to inactivate the enzymatical activity of NADPH oxidases (22, 23). As for the decrease in transcriptional levels of NADPH oxidases, we could speculate that the shift of sphingolipids in the OE could impact signaling cascades influencing promoter regions of NOXs' genes. For instance, the 5'-region of the human NOX1 gene contains binding elements for signal transducers and activators of transcription (STATs), interferon regulatory factor (IRF) (3), which are regulated by different sphingolipids (20). Thus, via its C1P-lyase activity, overexpression of SMPDL3b might regulate the transcriptional levels of NADPH oxidases. Alternatively, SMPDL3b might be influencing NADPH oxidases through direct protein-protein interaction. Such hypothesis is worth examining in the future given that the two enzymes are transmembrane proteins with activity modulated by lipid-rafts (8, 24).

NADPH oxidases contribute to many normal physiological processes such as cell signaling, host defense, and metabolism (3). Nonetheless, this family of enzymes is involved in numerous ROS-derived pathologies of renal dysfunction targeting podocytes particularly. For instance, NOXs have their share in orchestrating diabetic nephropathy (3, 25–28). Moreover, NOXs are implicated in a plethora of podocyte injury models such as hyperhomocysteinemia (29), FSGS (30), renal hypertension (31), and renal inflammation (32). However, the role of NOXs in the context of renal RT remained unclear. It has been reported that NADPH oxidases mediated radiation insult in rat brain microvascular endothelial cells (33) and ROS production in radiation-induced senescent cells (34). Additionally, inactivation of both NOX4 and NOX5 abrogated radiation injury in human primary fibroblasts (35). While countless studies have investigated NOXs' implication in diverse podocyte injury models, our results are the first to show an upregulation of NADPH oxidases upon RT in these cells.

Our work also demonstrates the impact of pharmacologic inhibition of NOXs as an approach to reverse radiation. Reversal of the renal fibrosis, proteinuria, and systolic pressure through NOX inhibition reveals that this family of enzymes contributes to the pathophysiology observed in the glomeruli post-RT *in vivo*. Increased systolic pressure is indicative of renal impairment (1, 36). Kidneys are key players in regulating systemic blood pressure through the renin-angiotensin-aldosterone system (RAAS). It is well established that RAAS-inhibition via the angiotensin-converting enzyme (ACE) and angiotensinogen I (ATI) inhibitors alleviates the progression of many kidney diseases including radiation nephropathy (37). Elevated diastolic and systolic blood pressures were noted after 100 days of kidney irradiation in mice, which were further increased upon bilateral renal radiation, pointing out that radiation nephropathy has long-lasting functional and metabolic consequences (38). Administration of GKT in our irradiated C57BL6 mice showed a decrease in systolic blood pressure 26 weeks later compared to control mice. Molecularly, protein levels of NOX1 and NOX4 were upregulated within 24hrs in irradiated renal cortices. This reflects that oxidative stress generated by NADPH oxidases is an early radiation response in renal cells. Moreover, the administration of GKT alleviated the cleavage of caspase-3 upon radiation *in vivo*, thus conferring radioprotection to renal cells.

Ultimately, our results show that the crosstalk between SMPDL3b and oxidative stress is critical for predicting the response of podocytes to radiation injury.

Ionizing radiation can cause injury not only to targeted podocytes but also to neighboring non-targeted cells which exhibit similar molecular damages and disturbances in the oxidative metabolism. The spread of the insult happens essentially through intercellular mechanisms (2). Moreover, crosstalks have been identified between different renal cell types and these intercellular communications play major roles in both healthy and diseased glomeruli, implicating oxidative stress, systemic chronic inflammation, and perturbations in the RAAS (11, 39, 40). While many studies have investigated this interplay in the context of diabetes especially (40, 41), none have examined this relationship in irradiated kidneys.

Glomerular endothelial cells (GenC), are key players in the onset and progression of numerous kidney diseases including radiation injury. Radiation-induced oxidative stress in glomerular endothelial cells has been examined by another study from our group (42). Irradiated GenC showed increased NOX activity and superoxide anion generation. Silencing NOX1 using NOX1-specific siRNA mitigated oxidative stress and cellular injury. Additionally, mice treated with GKT showed decreased apoptotic glomerular endothelial cells 24hrs post-irradiation. Ultimately, molecular interactions between glomerular endothelial cells and podocytes should be evaluated in the context of irradiation, ideally in a co-culture model or *in vivo*.

## Conclusion

Radiation nephropathy remains one of the main hurdles faced by patients towards their path to full recovery. Albeit remarkable advancements in radiation dose delivery techniques, damages to healthy neighboring renal tissues still occur. Our study establishes a radioprotective role of SMPDL3b in mitigating injury in podocytes in a ROS-dependent manner. Furthermore, our work unmasked a crosstalk between the lipid-modifying enzyme and the NADPH oxidases. Additional research is warranted to understand the mechanisms behind this crosstalk. Deciphering these events on the physiological and molecular levels might help unfold future therapeutic aspects in treating the radiation-induced nephrotoxicity. Therefore, proposing SMPDL3b as a novel therapeutic target holds major clinical implications given the canonical importance of podocytes in regulating renal function.

## Data Availability Statement

The original contributions presented in the study are included in the article/supplementary material, further inquiries can be directed to the corresponding author/s.

## Ethics Statement

The animal study was reviewed and by the Institutional Animal Care and Use Committee (IACUC) of American University of Beirut.

## Author Contributions

YZ, AF, BM, AE, and PA designed the research. PA and YZ analyzed the data and wrote the paper. PA, MF, MM, TY, and AD performed the research. All authors contributed to editing the paper.

## Funding

Funding was provided by NIH/NCI PQ12 1R01CA227493-01, and Medical Practice Plan Fund from the American University of Beirut. Research in Dr. Alessia Fornoni's laboratory is supported by the NIH grants R01DK117599, R01DK104753, R01CA227493, U54DK083912, UM1DK100846, U01DK116101 and UL1TR000460 (Miami Clinical Translational Science Institute).

## Conflict of Interest

AF is an inventor on pending or issued patents (PCT/US11/56272, PCT/US12/62594, PCT/US2019/041730, PCT/US2019/032215, PCT/US13/36484, and PCT 62/674,897) aimed to diagnosing or treating proteinuric kidney diseases and stands to gain royalties from their 20 future commercialization of these patents. AF is Vice-President of L&F Health LLC and is a consultant for ZyVersa Therapeutics, Inc. ZyVersa Therapeutics, Inc has licensed worldwide rights to develop and commercialize hydroxypropyl-beta-cyclodextrin from LF Research for the treatment of kidney disease. AF is the founder of LipoNexT LLC. AF is supported by Hoffman-La Roche and by Boehringer Ingelheim. The remaining authors declare that the research was conducted in the absence of any commercial or financial relationships that could be construed as a potential conflict of interest.

## Publisher's Note

All claims expressed in this article are solely those of the authors and do not necessarily represent those of their affiliated organizations, or those of the publisher, the editors and the reviewers. Any product that may be evaluated in this article, or claim that may be made by its manufacturer, is not guaranteed or endorsed by the publisher.

## Abbreviations

C1P: ceramide-1-phosphate
DSBs: double-strand breaks
FGSG: focal segmental glomerulosclerosis
GenC: glomerular endothelial cells
γ-H2AX: phosphorylated histone H2AX
NAC: N-acetylcysteine
NOXs: NADPH oxidases
OE: overexpressors of SMPDL3b
ROS: reactive oxygen species
RT: radiotherapy
SMPDL3b: sphingomyelinase acid phosphodiesterase like 3b
WT: wild type
RAAS: renin-angiotensin-aldosterone system.
